# A Closed Loop Stimuli-Responsive Concanavalin A-Loaded Chitosan–Pluronic Hydrogel for Glucose-Responsive Delivery of Short-Acting Insulin Prototyped in RIN-5F Pancreatic Cells

**DOI:** 10.3390/biomedicines11092545

**Published:** 2023-09-15

**Authors:** Shazia Mansoor, Samson A. Adeyemi, Pierre P. D. Kondiah, Yahya E. Choonara

**Affiliations:** Wits Advanced Drug Delivery Platform Research Unit, Department of Pharmacy and Pharmacology, School of Therapeutic Science, Faculty of Health Sciences, University of the Witwatersrand, Johannesburg 2093, South Africa; 707825@students.wits.ac.za (S.M.); samson.adeyemi@wits.ac.za (S.A.A.); pierre.kondiah@wits.ac.za (P.P.D.K.)

**Keywords:** concanavalin A, closed loop, hydrogel, insulin delivery, thermosensitive, glucose responsive

## Abstract

The optimal treatment of diabetes (in particular, type 1 diabetes—T1D) remains a challenge. Closed-loop systems (implants/inserts) provide significant advantages for glucose responsivity and providing real-time sustained release of rapid-acting insulin. Concanavalin A (ConA), a glucose affinity agent, has been used to design closed-loop insulin delivery systems but not without significant risk of leakage of ConA from the matrices and poor mechanical strength of the hydrogels impacting longevity and control of insulin release. Therefore, this work focused on employing a thermoresponsive co-forming matrix between Pluronic F-127 (PL) and structurally robust chitosan (CHT) via EDC/NHS coupling (i.e., covalent linkage of -NH_2_ from CHT and ConA to the -COOH of PL). The system was characterized for its chemical structure stability and integrity (FTIR, XRD and TGA), injectability, rheological parameters and hydrogel morphology (Texture Analysis, Elastosens TM Bio2 and SEM). The prepared hydrogels demonstrated shear-thinning for injectability with a maximum force of 4.9 ± 8.3 N in a 26G needle with sol–gel transitioning from 25 to 38 °C. The apparent yield stress value of the hydrogel was determined to be 67.47 Pa. The insulin loading efficiency within the hydrogel matrix was calculated to be 46.8%. Insulin release studies revealed glucose responsiveness in simulated glycemic media (4 and 10 mg/mL) over 7 days (97%) (305 nm via fluorescence spectrophotometry). The MTT studies were performed over 72 h on RIN-5F pancreatic cells with viability results >80%. Results revealed that the thermoresponsive hydrogel is a promising alternative to current closed-loop insulin delivery systems.

## 1. Introduction

Patients diagnosed with type 1 (or severe type 2) diabetes are dependent on multi-dose exogenous insulin administration. Β-cells within the pancreas are unable to produce insulin (particularly in T1D), and in severe cases of T2D, they cannot produce sufficient insulin to maintain blood glucose levels. A challenge is that short-acting insulin must be injected often and is unstable for oral administration due to various GIT factors that include enzymatic degradation. Hence, smarter biomaterials such as injectable hydrogels in combination with natural and/or synthetic polymers have been explored as alternative delivery systems to provide sustained release of insulin [1].

In particular, stimuli-responsive injectable hydrogels can be designed as closed-loop systems [2] to enable the delivery of therapeutic proteins (e.g., insulin) in real time to target sites in a dose-responsive manner. An advantage of such closed loop injectable systems is that they do not require surgical insertion of device (e.g., indwelling insulin pumps) and can provide minimally invasive delivery of therapeutics [3]. Closed-loop systems have been reported in numerous studies to provide rigid control of insulin release with a positive outcome for diabetic patients [4]. Closed-loop systems include insulin infusion pumps and chemically controlled systems that enable a feedback loop of insulin delivery as the body requires. Among the chemically responsive closed loop systems, glucose oxidase (GOx) and phenylboronic acid (PBA) have been extensively researched. These two approaches are limited by a lag response time and pH specificity, respectively [5].

**Figure 1:**
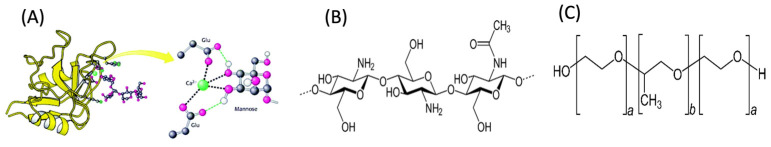
(**A**) A tetramer of jack bean concanavalin A’s crystal structure with C-type carbohydrate-binding domain with binding to glucose and mannose, respectively [13]. (**B**) Chemical structure of chitosan. (**C**) Chemical structure of tri block polymer, Pluronic F-127 that has block lengths of a = 101 and b = 56.

The use of biomaterials has gained tremendous attention and gains in the design of novel drug delivery systems. Concanavalin A (ConA) (a type of lectin) is an excellent material to design glucose-responsive hydrogels (Figure 1A). ConA was shown to maintain basal and bolus insulin secretion in response to various glucose concentrations with minimal fluctuation in blood glucose levels and having it imbibed within a thermoresponsive hydrogel provides an added benefit to controlling insulin release, preventing ConA leakage, poor stability, poor control of insulin [6,7] and improving injectability [8,9].

Pluronic F-127 (PL) (Figure 1C), a linear glucan-based triblock polymer, was used as the sol–gel transitioning matrix above its lower critical solution temperature (LCST) between 25 and 37 °C [10]. This approach is unique to other dextran-based branched glucans that have been extensively explored to prepare with ConA. PL provides the added advantage of preventing premature leakage of ConA from the system due to favorable covalent interactions during carboxylation of PL and EDC/NHS activation/coupling. In addition, chitosan (CHT) is a natural cationic carbohydrate polymer constituting α-1,4-linked 2-amino-2-deoxy-D-glucose (N-acetyl glucosamine) with protonation of free amino (-NH_2_) groups [11]. Therefore, this study focused on the design of an injectable (minimally invasive) chitosan–Pluronic F127–ConA (CHT-PL–ConA) thermoresponsive hydrogel for the specialized delivery of insulin as an alternative closed-loop system for T1D. CHT was used as a matrix co-former to increase the mechanical strength of the thermoresponsive hydrogel and was crosslinked (EDC/NHS coupling) with the PL during matrix synthesis. The crosslinking of CHT was to enhance the controlled release of insulin and improve the ConA sensitivity to glucose [12]. The system was characterized for its physiochemical and physicomechanical properties as well as the analyses of the sustained release of insulin over 7 days followed by in vitro cell testing using an MTT assay.

## 2. Materials and Methods

### 2.1. Materials

Concanavalin A (ConA) (104 kDa), Pluronic F-127 (12.5 kDa), high molecular weight Chitosan (CHT) (310–375 kDa, (85–95% deacetylation degree)), 1-ethyl-3-(3-dimethylaminopropyl)-carbodiimide (EDC), N-hydroxy succinimide (NHS), succinic anhydride, hydrochloric acid (HCL), 4-dimethyl aminopyridine (DMAP), triethylamine (TEA), 1,4 dioxane, sodium hydroxide (NaOH), 1,4-dioxane, cellulose dialysis membrane (MWCO = 14,000), phosphate-buffered saline (PBS), were all purchased from Sigma-Aldrich (St. Louis, MO, USA). Rapid-acting insulin analogue was obtained from Eli Lily and Company (Indianapolis, IN, USA). The morpholineethanesulfonic acid (MES), magnesium chloride (MgCl_2_), calcium chloride (CaCl_2_) and manganese chloride (MnCl_2_) were purchased from Merck (Pty) Ltd., (Modderfontein, South Africa). RIN-5 cells were acquired from Cellonex (Johannesburg, South Africa). RPMI, pen-strep, fetal bovine serum (FBS) were purchased from Sep Sci (Johannesburg, South Africa). MTT toxicity kit was procured from Sigma Aldrich (St. Louis, MO, USA). All other reagents obtained were of analytical grade and used without further purification.

### 2.2. Synthesis of the Insulin-Loaded PL–CHT–ConA Stimulus-Responsive Hydrogel

Preparation of the chitosan–Pluronic F127–concanavalin A (PL–CHT–ConA) stimuli (thermo/glucose)-responsive hydrogel was designed to deliver the short-acting insulin within a chemically controlled closed-loop system over a period of 7 days. Firstly, the CHT-monocarboxylated PL copolymer was synthesized according to Chung and co-workers (2005) [14] with modifications. Briefly, Pluronic F-127 (14 g) was added to DMAP (62 mg), succinic anhydride (50 mg), TEA 50 (µL) and 1, 4 dioxane (15 mL). The carboxylation of PL took place via esterification with succinic anhydride. Thereafter, the mixture was stirred using a glass rod and placed on a heated magnetic stirrer set at between 25 and 30 °C for 24 h. To achieve constant temperature, the glass beaker was placed in a temperature-monitored silicone bath. The mixture was then removed and dialyzed overnight with distilled water to remove any excess succinic anhydride [15]. Once dialysis was completed, EDC (232 mg) and NHS (64 mg), dissolved in 0.1 M MES buffer at pH 6, was added dropwise and left to stir for 4 h to activate the functional groups. Activated ConA (800 µL) in PBS containing MgCl_2_, CaCl_2_ and MnCl_2_ salts were added dropwise while stirring [15,16]. Lastly, CHT (85–95% deacetylation degree) dissolved in 0.1 N HCl was added to the mixture and left in an orbital shaker (37 °C; 24 h) [17]. The product was then dialyzed and placed in a vacuum oven overnight to cure. In addition, following the method described by Zhao et al. (2017) [18] with modifications, the preparation of the insulin-loaded hydrogel involved simple mixing (vortex) and self-assembling processes in which the preformed PL–CHT–ConA crosslinked hydrogel was dissolved in PBS (pH 7.4) with insulin concentration of 0.6 or 10 mg/mL. Unbound insulin that adhered to the surface of the hydrogel was washed away in PBS at pH 7.4 for 2 h in an orbital shaker. The formulation was further shaken in an orbital shaker (100 r/min, 37 °C) for 9 h prior to further characterization. 

### 2.3. Elucidation of the Physicochemical and Morphological Stability of the Stimuli-Responsive PL–CHT–ConA Hydrogel

Fourier transform infrared (FTIR) spectroscopy was performed on individual components and the resultant dual-responsive hydrogel to evaluate the chemical structural stability. The FTIR spectra were recorded on a Perkin Elmer Spectrum 2000 FTIR spectrometer with an MIRTGS detector, using an ATR-FTIR diamond crystal internal reflection element (PerkinElmer Spectrum 100, Llantrisant, Wales, UK). Samples were analyzed at a wavelength range of 4000–650 cm^−1^ with a resolution of 4 cm^−1^, 64 scans per spectrum and a constant pressure of 120 psi. The hydrogel system was lyophilized and along with the pristine polymers was analyzed using X-ray diffraction (XRD) using a benchtop Rigaku MiniFlex fitted with a high-speed silicon strip detector and operating at 600 W X-ray source for high-resolution scanning (Tokyo, Japan). XRD was performed to establish the crystalline or amorphous nature of the components or any changes to the hydrogel matrix. 

A thermogravimetric analyzer (TGA 4000, PerkinElmer, Llantrisant, Wales, UK) was used to analyze the thermal decomposition of the CHC-PL–ConA hydrogel matrix and its components, over time. Samples (15–20 mg) were placed in a ceramic crucible and then under a constant nitrogen gas atmosphere. The experiments were run at 10 °C/min from 70 to 800 °C, after which the thermograms were obtained and analyzed (% weight vs. temperature graph) to determine the thermal stability of the hydrogel system.

The thermophysical characteristics of the fabricated delivery system and its components were determined using differential scanning calorimetry (DSC) (Mettler Toledo, DSC-1, STARe System, Swchwerzenback, ZH, Switzerland). Reagents were weighed between 3 and 10 mg and sealed within perforated aluminum crucibles. Thereafter, they were heated from 0 to 400 °C at a heating rate of 10 °C/min under constant N_2_ purging. The data were plotted as heat flow against temperature.

### 2.4. Determination of the Physicomechanical Properties of the PL–CHT–ConA Stimuli-Responsive Hydrogel

#### 2.4.1. Textural Profiling Analysis for System Injectability

To assess the injectability (via sol–gel transitioning) of the hydrogel matrix, textural profile analysis was carried out using a texture analyzer (TA. XTplus Texture Analyzer, Stable Micro Systems, Surrey, UK). Once injected, the delivery system should transition to a gel state at body temperature (37 °C). Injectability tests were performed in compression mode employing a 1 mL Luer-lock syringe with either a 21 G or 26 G needle attached, at a calibration force capacity of 2 kg, a test speed of 20 mm/s and a return distance of 50 mm. The sample was run at a test speed of 1 mm/s and distance of 40 mm using a load-cell of 50 kg. Analysis was undertaken in triplicate with the average of the force (N) required to inject the hydrogel through the needles demonstrated with a corresponding standard deviation [19,20]. 

#### 2.4.2. Dynamic Nondestructive Sol–Gel Transitioning Analysis of the PL–CHT–ConA Hydrogel

An ElastoSensTM Bio2 (Rheolution Instruments, Quebec, QC, Canada) was used for dynamic nondestructive rheological analysis of the sol–gel transitioning properties of the hydrogel matrix. This was performed to determine the optimal sol–gel transition temperature of the matrix and the gel–glucose responsiveness (G′) in response to varying simulated glucose levels. These techniques measured G′ and G″ as a function of temperature in real time or time at 37 °C. Experiments were carried out in triplicate; samples were transferred to holders wherein gentle mechanical vibration was applied with laser detection to avoid contact with the sample and remove any bias from physically disturbed molecular arrangements.

#### 2.4.3. Temperature-Dependent Flow Analysis of the PL–CHT–ConA Hydrogel Matrix

Important flow characteristics of the delivery system were analyzed by using a Haake Modular Advanced Rheometer System (ll) (MARS) with a C35/1° titanium rotor sensor (ThermoFisher Scientific, Karlsruhe, Germany). Analysis was carried out on the delivery system to determine the viscosity (ƞ*) of the system. These parameters provide insight into the liquid- and solid-like characteristics of the hydrogel in response to temperature. Rheological measurements were evaluated using a cone–plate apparatus with a diameter of 35 mm and cone angle of 1°.

### 2.5. Surface Morphology Analysis of the PL–CHT–ConA Hydogel Matrix

Samples of the hydrogel matrix were air dried at room temperature (25 °C) and mounted on aluminum stubs with carbon double-sided tape. This was sputter coated with gold–palladium compound employing an SPI sputter coater (SPI Module TM sputter-coater and control unit, West Chester, PA, USA). The morphology of the hydrogel system was determined using an FE SEM (Zeiss Sigma, Jena, Germany) at an EXTVT = 2.5, 113 current and EHT accelerating voltage of 4 kV. 

### 2.6. Determination of In Vitro Glucose Responsivity and Insulin Release from the PL–CHT–ConA Hydrogel Matrix

To carry out insulin release studies via UV-vis spectrophotometer, a wave scan was initially carried out to determine λ_max_. Thereafter, a calibration curve was established using a wavelength of 270 nm to determine the concentration of drug. Insulin release studies were carried out according to Lin et al. (2019) [15] with modifications. The formulated delivery system was blended with PBS and placed in dialysis tubing (2 mL). The dialysis tubing and was then immersed in 100 mL of PBS at different glucose concentrations: glucose-free (control), at physiological blood glucose levels (400 mg/dL) and in a simulated hyperglycemic environment (1000 mg/dL), and placed in an orbital shaker set at 37 ± 0.5 °C, 100 r/min. The release of insulin was achieved in response to stepwise changes in glucose concentration (4 and 10 mg/mL). At appropriate time intervals (0, 15, 30, 45 min and 1, 2, 4, 8, 12, 24, 48, 72, 168 h), 1000 μL of the released medium was sampled and the insulin concentration was quantitatively measured via UV-vis at a wavelength of 270 nm (N = 3). The assay was continued after replacement with an equal volume of fresh release medium to maintain a constant volume. The percentage of insulin release was determined by using the cumulative release as per Equation (1).

Cumulative Release (%) = ((Total amt of insulin released per time-point)/ (Total amt of insulin incorporated into gel)) × 100(1)

### 2.7. Insulin Stability Analysis within the PL–CHT–ConA Hydrogel Matrix

The structural stability of released insulin was determined using fluorescence (FL) spectrophotometry. Samples of insulin-loaded hydrogel were analyzed as per the release study experimental setup after 168 h and tested at an excitation wavelength of 276 nm and an emission wavelength of 305 nm. A standard insulin solution was set to 0.02 mg/mL, adapted with modifications from Bai et al. (2018) [21]. Samples were run in triplicate and the data were the mean obtained.

### 2.8. Cytotoxic Assay of the PL–CHT–ConA Hydrogel Matrix 

In vitro cytotoxicity investigations are a crucial parameter to establish prior to the progression of animal studies [22]. Cell viability studies were undertaken to evaluate the cytotoxic effects of the dual hydrogel using the 3-(4,5-Dimethylthiazol-2-yl)-2,5-diphenyltetrazolium bromide (MTT) assay kit. The MTT assay provides an insight into cell viability via a color change of yellow to purple, which is present in the mitochondria of active cells. This assay was carried out using an in vitro toxicology assay kit on RIN-5F, as per the protocol provided. T75 culture flasks were used to grow the cells at 37 °C humidified air, 5% carbon dioxide and in RPMI media.

The insulin loaded hydrogel was added to the 96-well plates at varying concentrations established from in vitro insulin release studies, along with the negative and positive control of plain cells and 5 fluorouracil. The unloaded hydrogel was also added at a volume of 10 µL and left for 72 h. After the incubation period, the medium was removed and MTT solution (10 µL) was added to the wells. The cells were incubated for 4 h to allow for the formation of blue formazan crystals. The MTT solution was then replaced with solubilizing buffer (100 μL/well), and cells were incubated overnight. Absorbance was measured at 570 nm in a Synergy HT multiwell microplate reader (BioTek^®^ Instruments, Winooski, VT, USA). The percentage cell viability was calculated by comparing the mean optical density (OD) of the treated cells with the blank wells (medium with no cells) and nontreated control cells using Equation (2):

Cell Viability % = (Avg. Absorbance of the samples/Blank) ×100(2)

## 3. Results and Discussion

### 3.1. Preparation of PL–CHT–ConA Hydrogel Matrix Dual-Responsive Hydrogel

**Figure 2:**
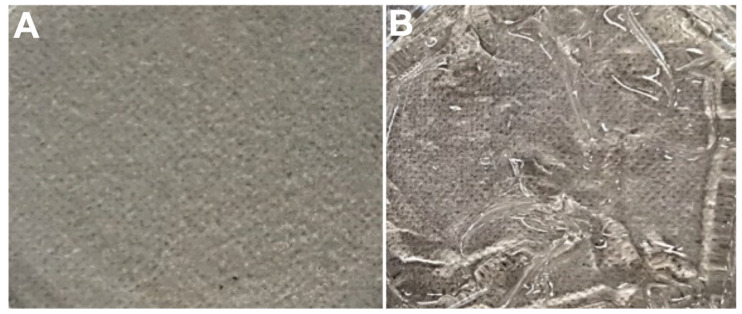
Photographic image of (**A**) the hydrogel air-dried at room temperature in a Petri dish and (**B**) rehydrated version of dual-responsive hydrogel, demonstrating the swelling properties of synthesized formulation.

The synthesis of this closed-loop, dual-responsive hydrogel demonstrated the application of chemically crosslinked polymers and the lectin protein ConA, using EDC/NHS coupling by exploring the covalent interaction of –COOH groups to –NH_2_ groups on HMW chitosan and ConA [15]. The reaction entails the formation of an intermediate active ester (the product of condensation of the carboxylic group and NHS), which further reacts with the amine functional group to yield an amide bond [15]. ConA can selectively bind to glucose molecules and as such are usually employed for the fabrication of optical fiber glucose sensors, which have preferential sensitivity to glucose solutions [23]. The hydrogel undergoes sol–gel transition, as seen in Figure 2 below. The formulated dual-responsive system is an example of an idealized therapy for the treatment of diabetes mellitus (DM). 

### 3.2. Determination of Structure and Purity of Pristine Polymers and Formulated PL–CHT–ConA Hydrogel Matrix

**Figure 3:**
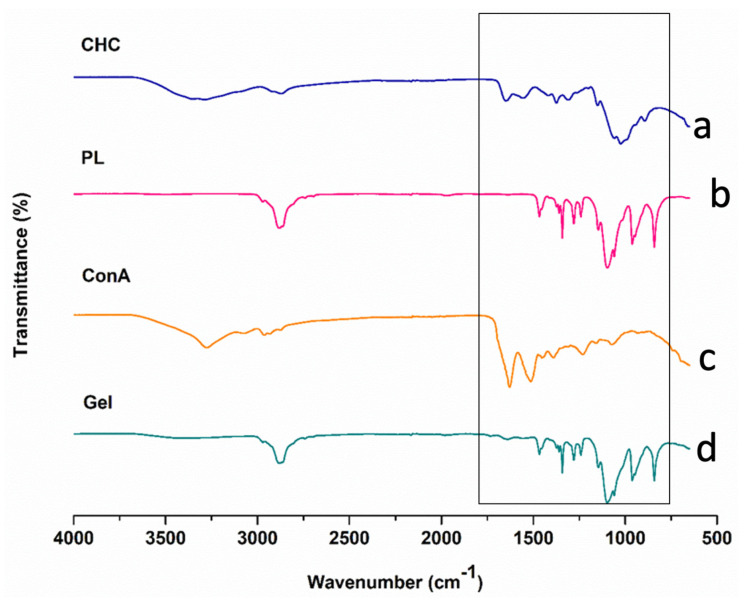
FTIR spectra of (a) CHC, (b) PL, (c) ConA and (d) the formulated dual-responsive hydrogel.

As presented in Figure 3, FTIR was carried out to analyze the reactions of chitosan to Pluronic and the addition of ConA to form the synthesized hydrogel. The pristine polymer, high molecular weight CHT displayed characteristic broad absorption peaks at 3286 cm^−1^ (OH and amine stretching vibrations), 2874 cm^−1^ (C–H stretching), 1650 cm^−1^ (amide I C=O stretching), 1542 cm^−1^ (amide II stretch), 1380 cm^−1^ (amide III bending), 1150 cm^−1^ and 1023 cm^−1^ (antisymmetric stretching of the C–O–C bridge and skeletal vibrations around the C–O stretching feature of its saccharide chemical structure) and 894 cm^−1^ (pyranose ring). These were consistent with CHT peaks observed in the literature [24,25]. PL demonstrated characteristic IR peaks at 2882 cm^−1^ (C–H aliphatic stretch aliphatic), 1343 cm^−1^ (in-plane O-H bend) and 1100 cm^−1^ (C–O stretching vibrations) as described by Karolewicz et al., (2017) [26]. The protein lectin ConA displayed peaks at 3276 cm^−1^ (OH or Backbone NH stretching), 2962 cm^−1^ (CH_2_ stretching vibrations), 1628 cm^−1^ (amide I and β-sheet of protein) 1614 cm^−1^ (C=O) and 1230 cm^−1^ (amide III band), which correspond to ConA IR spectra as described by [27,28]. 

The formulated PL–CHT–ConA hydrogel peaks were observed at 3401 cm^−1^ (N–H stretching from the pristine polymer CHT and ConA), 2878 cm^−1^ (C-H stretching), 1640 cm^−1^ (corresponding to the amide I of CHT and the β-sheet of ConA), 1465 cm^−1^ (C–H bending), 1341 cm^−1^ (O–H bending from PL), 1098 cm^−1^ and 1059 cm^−1^ (C–N stretching amine); thus, it is evident that the interaction of -NH groups of CHT and ConA with the –COOH groups of PL in the conjugated nanosystem accounted for the successful formulation of the hydrogel [29,30]. 

### 3.3. X-ray Diffraction Studies on Pristine Polymers and PL–CHT–ConA Hydrogel Matrix

**Figure 4:**
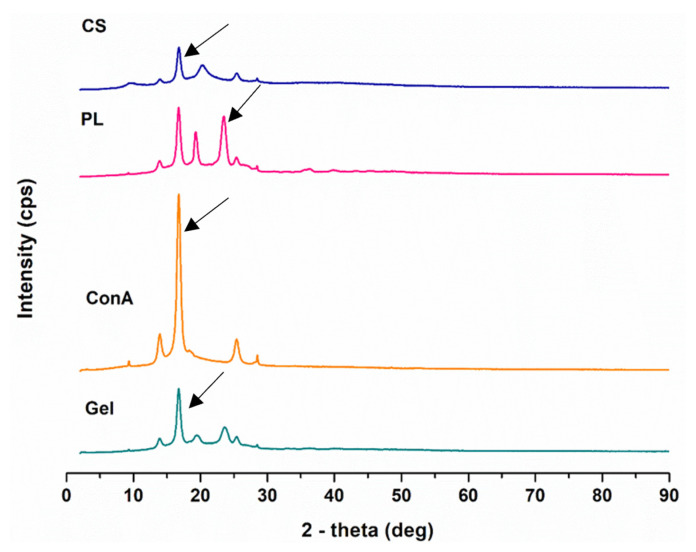
XRD peaks of the pristine polymers, ConA and formulated hydrogel.

X-ray diffraction analyses were conducted to further analyze the amorphous or crystalline structure of the synthesized hydrogel and its components. As per XRD analysis, materials can be either crystalline, semicrystalline or amorphous [30]. CHT is a well-known semicrystalline compound that possesses both weak and strong crystallinity. This is due to the formation of hydrogen bonds between the amino and hydroxyl groups that are consistently present in the CHT structure. The characteristic XRD patterns of pristine CHT show two distinct diffraction peaks at 2θ of 9.55° and 20° with the later demonstrating CHT crystalline form (Figure 4) [31,32]. PL is also a semicrystalline polymer with crystalline PEO layers and PEO and PPO forming amorphous layers. PL revealed distinct peaks at diffraction angle at 2θ of 14°, 19.2° and 24° [33,34]. ConA displayed XRD peaks at 2θ of 14°, a sharp crystalline peak at 16° and a peak at 26°. Accordingly, the formulated hydrogel displayed multiple crystalline and amorphous peaks with lower intensities possibly due to the interactions of carboxyl and amino groups, at 9.55° from CHT, 19.2° from PL and 26° from ConA.

### 3.4. Determination of Mass Change in PL–CHT–ConA Hydrogel System and Components 

**Figure 5:**
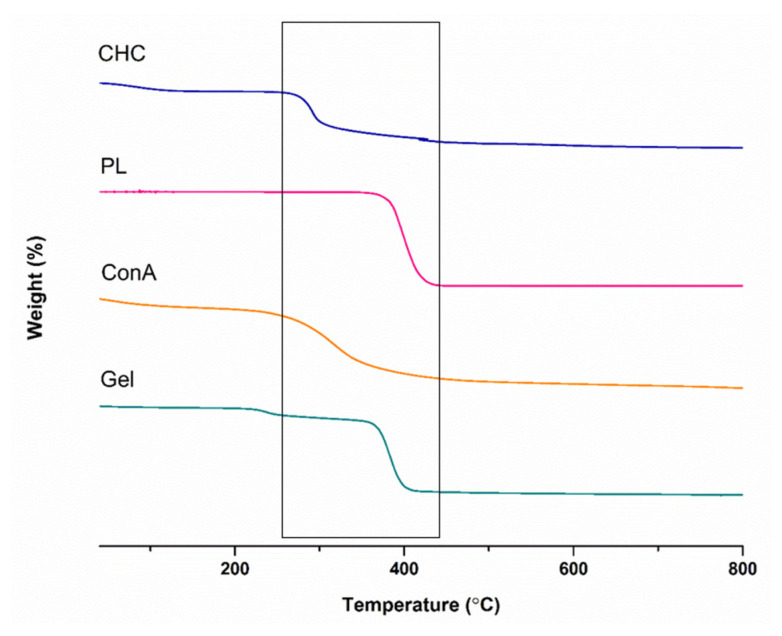
The TGA curve of the pristine polymers, ConA and the dual-responsive hydrogel, illustrating the weight change % over the 70–800 °C temperature range.

TGA analysis was used to study the relationship between temperature and weight loss as it relates to thermal qualities such as stability and decomposition. Pristine polymers, the glucose-responsive compound ConA, and the dual-responsive PL–CHT–ConA hydrogel were studied in this investigation. The range of the temperature for the weight-loss curve was 70 to 800 °C in Figure 5. The decomposition of the pristine polymer CHC occurred at the 300 °C region. This corresponds to the degradation of the CHT main chain [35]. The mass change or degradation of PL was observed in a single-step at the range of 350–410 °C. These results are consistent with previous investigations [36,37]. ConA exhibited a gradual weight-loss % between 260–400 °C [38]. The thermal decomposition of the formulated hydrogel displayed a slight weight-loss around 250 °C possibly due to chitosan and ConA amino group interactions and a secondary rapid decomposition at 360 °C to 400 °C because of increasing hydrogen bond formations between carboxylic groups and unsubstituted amino groups, in accordance with the literature [30,39].

**Figure 6:**
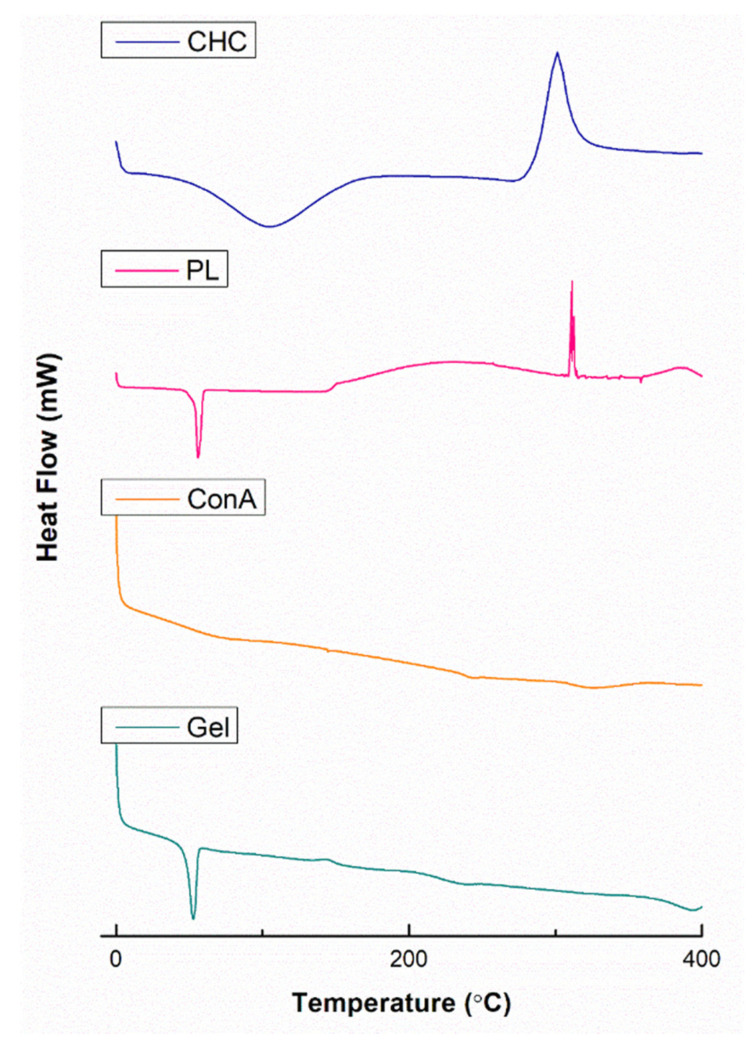
DSC depicting phase change of pristine polymers, ConA and the synthesized hydrogel over temperature range 0–400 °C.

In contrast to TGA, in which weight change is measured, DSC determines the amount of energy absorbed or released by a sample and its corresponding phase change during heating or cooling, as seen in Figure 6. In the thermogram of CHC, two curves were observed. The endothermic curve at 104 °C, which illustrated the phase change of CHC from a solid state to a rubbery, soft state. The exothermic curve at 301 °C represents degradation of CHC while releasing 2218 mJ of energy [32]. The melting point for PL was seen at 56.65 °C while absorbing 1424.66 mJ of energy and its complete degradation taking place at 310 °C [30]. Thermodynamic data for ConA show sharp melting, possibly due to its extreme temperature sensitivity as a protein. The literature suggests melting at around 70–82 °C with an enthalpy of 252 kJmol^−1^ [40]. The hydrogel demonstrated a single endothermic peak at 52.86 °C changing phase into a soft substance and gaining 217.37 mJ of energy. This shift in endothermic temperature is suggestive of covalent bonding between CHC, ConA and PL, indicating formation of the hydrogel [30,33].

### 3.5. Determination of the Viscoelastic Characteristics of the Thermo/Glucose PL–CHT–ConA Responsive Hydrogel

#### 3.5.1. Determination of Force Required for Injectability of PL–CHT–ConA Hydrogel

**Figure 7:**
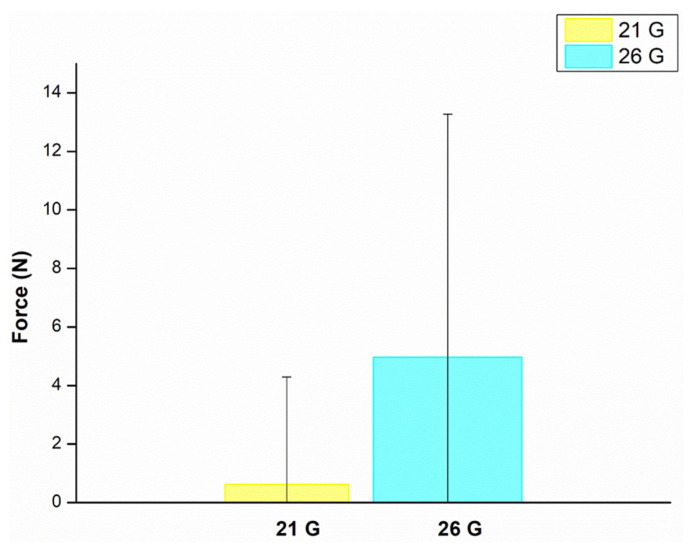
Maximum injection force determination (N) demonstrating the injectability of the prepared hydrogel employing two needle gauges.

Injectability is the measure of force required to administer a drug in a uniform manner, while keeping the needle free of clogging [41]. The injectability of the hydrogel was shown to be possible through both the 21 G and 26 G needles with a maximum force of 4.9 ± 8.3 N in the 26 G needle and a maximum force 0.6 ± 3.6 N in the 21 G needle (Figure 7). The parameter affecting injectability is the gauge of needles: the larger the gauge, the more force needed. The recommended value for the maximum force exerted manually is 30 N while injectability force for minimal physical distress is 10 N. Therefore, the hydrogel is within these limits and is suitable for injectable administration [20,42].

#### 3.5.2. ElastoSensTM Bio2 Thermoresponsiveness Studies of PL–CHT–ConA Hydrogel

**Figure 8:**
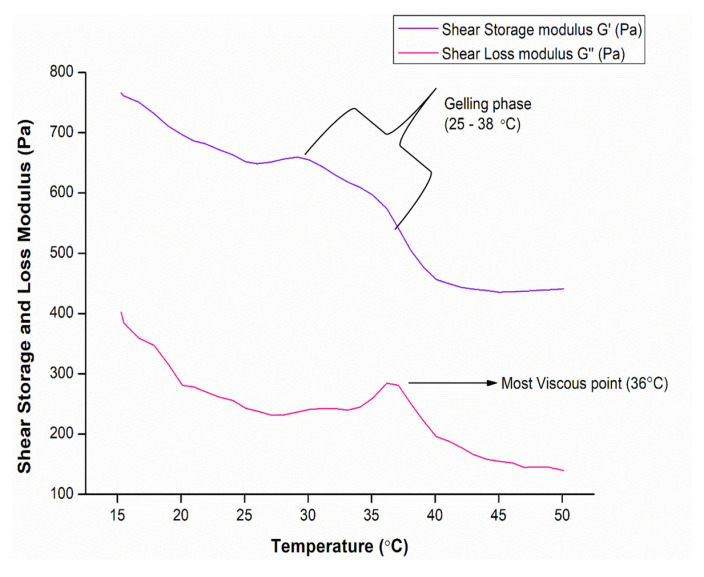
Thermogelation behavior of hydrogel employing ElastoSensTM Bio2 sheer stress vs. temperature.

The ElastoSensTM Bio2 was employed to investigate the temperature at which the formulated hydrogel transitions from sol to gel. This was an important parameter to consider, as the hydrogel was designed to undergo gelling once injected into the body. G′ is a measure of the stiffness of the gel, while G″ provides an indication of the softness of the gel. Herein, we generated the G′ and G″ curves, which demonstrated an increase in the storage modulus yielding the gelling phase of 25–38 °C and a peak in the loss modulus demonstrating the most viscous point of the hydrogel at 36 °C (Figure 8). This confirmed that the hydrogel does undergo sol–gel transition when injected at body temperature, thereby making it an ideal candidate for drug delivery or tissue engineering [43].

#### 3.5.3. Determination of G′ and G″ of Hydrogel System

##### Temperature Ramp and Viscoelasticity 

**Figure 9:**
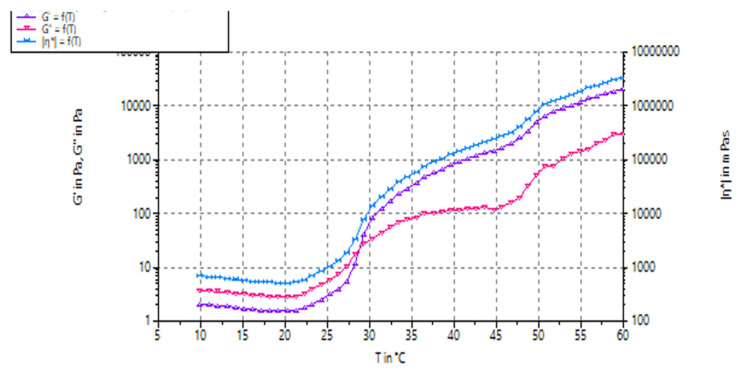
The storage (G′), loss modulus (G″) and viscosity (ƞ*) of the dual-responsive hydrogel. The gelling temperature of the hydrogel is where G′ and G″ cross.

Viscoelasticity is an important property of closed-loop hydrogels for insulin delivery. This property allows the hydrogel to respond to changes in glucose levels and release insulin, accordingly, thus mimicking the function of a healthy pancreas. It also ensures sustained release of insulin over an extended period, reducing the need for frequent injections. Thus, further mechanical properties of the dual-responsive hydrogel were examined by employing the rheometer and studying the G′, storage modulus and G″, loss modulus and viscosity (ƞ*) over a temperature range of 10–60 °C. The graph (Figure 9) demonstrated G′, G″ and ƞ* remaining below 10 Pa initially until 22.5 °C, representing a liquid state. Thereafter, a concurrent increase in G′, G″ and ƞ* was observed until sol–gel transition occurred where G′ and G″ crossover at approximately 28 °C and G′ > G″ after 30 °C, when Pa increases rapidly. Thus, the hydrogel is more viscoelastic than liquid, as corroborated by the ƞ* curve. This indicates that the network matrix was completed through hydrophobic interactions of the PPO groups of PL and hydrophilic groups of CHC and ConA. These changes may be possible due to -OH functional groups present at the end chains; thus, sol–gel transition may take place easily. The hydrogel is thus thermoresponsive. This is advantageous for long-term delivery of drugs, as it overcomes the problem of multiple daily self-administered insulin injections.

##### Rheometer—Yield Stress and Shear Viscosity Determination of PL–CHT–ConA Hydrogel

**Figure 10:**
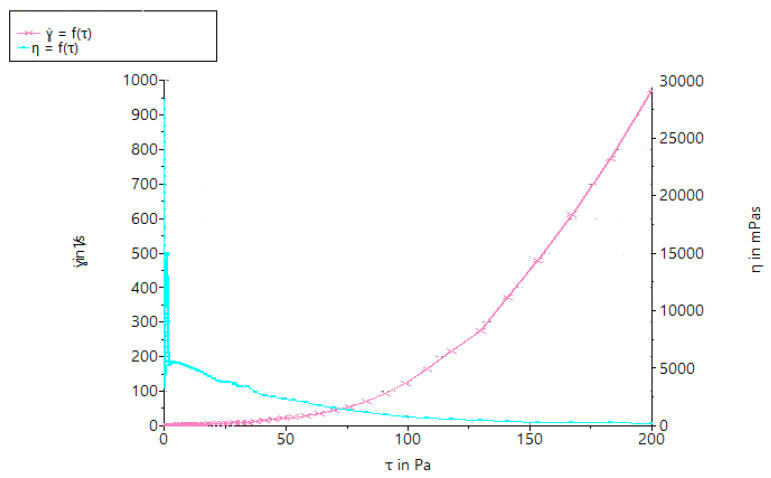
Yield stress and shear rate determination of the hydrogel.

Yield stress or yield point is the lowest shear stress value above which a material will behave like a fluid and below which the material will act like a solid. By adjusting the yield point of a sample, the force that is needed for the sample to start flowing out can be simulated. This phenomenon is commonly observed in non-Newtonian fluids, where the viscosity changes with the shear rate. The apparent yield stress value of the hydrogel was determined and was found to be 67.47 Pa (Figure 10). This is the point at which the hydrogel begins to flow and is an important parameter for ease of injectability. The yield stress increased as the fluid substance became more viscous (ƞ) thus indicating that the hydrogel has high resistance to flow. The increase in yield stress can also be attributed to the formation of a more structured network within the fluid. Shear viscosity is the resistance of hydrogel flow during extrusion; hence, as the formulation is designed to be injectable, shear viscosity is a crucial factor to consider. Since shear viscosity is a function of shear stress over shear rate, we can observe that as the shear rate is increasing, the viscosity is decreasing, and a deformation change in the hydrogel is taking place as the force is applied, allowing for ease of injectability and corresponding to TA injectability analysis.

##### Oscillation Stress Sweep

**Figure 11:**
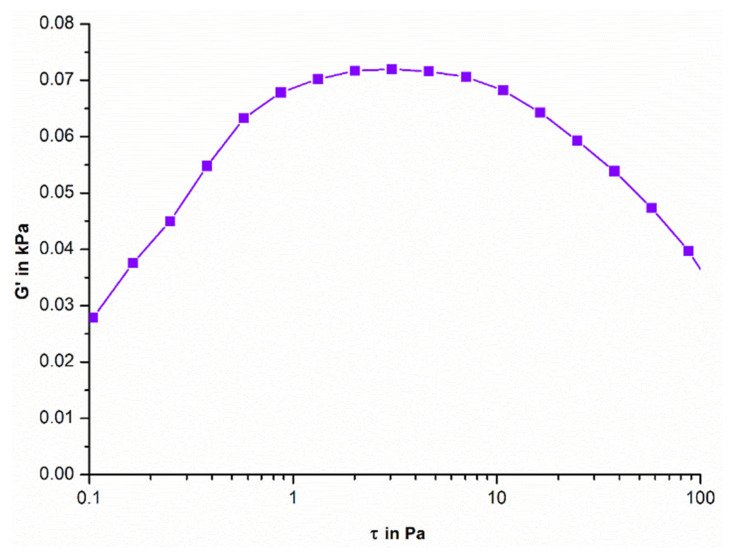
Oscillatory stress sweep of hydrogel.

An oscillation stress sweep is a rheological test that involves applying sinusoidal stress to a sample and measuring the resulting strain. It is an important tool for probing soft structures, allowing us to identify properties such as pickup or stability and predict drainage behavior. An initial increase in G′ is seen, which is related to a rise in the degree of intermolecular crosslinking within the formulation, which increases the mechanical strength of the formulation [44]. This correlates to ElastosensTM Bio2 results on which G′ was increasing and thus the stiffness of the hydrogel was increasing (Figure 11) indicating both glucose- and thermoresponsiveness of the formulated hydrogel. The viscoelastic region is where the response to stress is both elastic and viscous, indicating a complex material behavior, which can be seen from 0.87 Pa to 10.74 Pa. The yield stress value is 10.74 Pa and is denoted by the point at which the elastic modulus (G′) starts to decline, indicating structural deformation [20]. 

### 3.6. Surface Morphology Analysis (SEM) of Formulated PL–CHT–ConA Hydrogel

**Figure 12:**
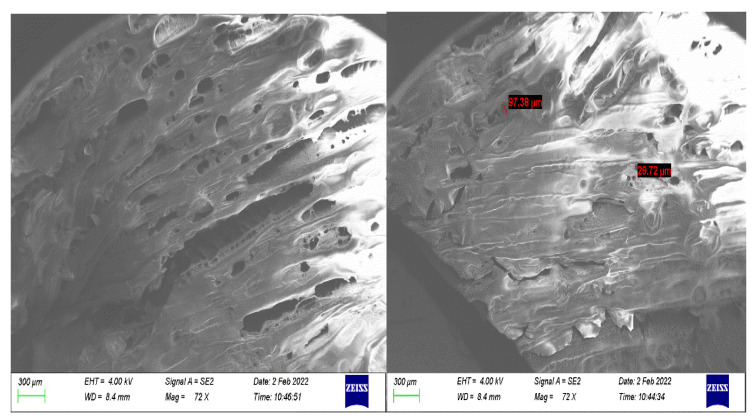
SEM micrograph of the surface of the dual-responsive hydrogel at a magnification of 72× and scale bar of 300 µm.

Hydrogels undergo sol–gel transition due to their porous structure, which allows them to swell and mimic the extracellular matrix (ECM) while also absorbing treatments and protecting them from external degradation within the body. Thus, it is important to observe their structure. The air-dried and sputter-coated hydrogel was micrographed, and the morphology was examined (Figure 12). The hydrogel demonstrated a smooth porous surface, with irregularly shaped and sized pores ranging from around 29.27 to 97.38 µm, with most pores being spherical. These examinations relate to the XRD diffractogram of the lyophilized hydrogel (Figure 4), which demonstrated multiple crystalline and amorphous peaks, thus correlating to its noted morphology.

### 3.7. In Vitro Insulin Release Kinetics from PL–CHT–ConA Hydrogel 

**Figure 13:**
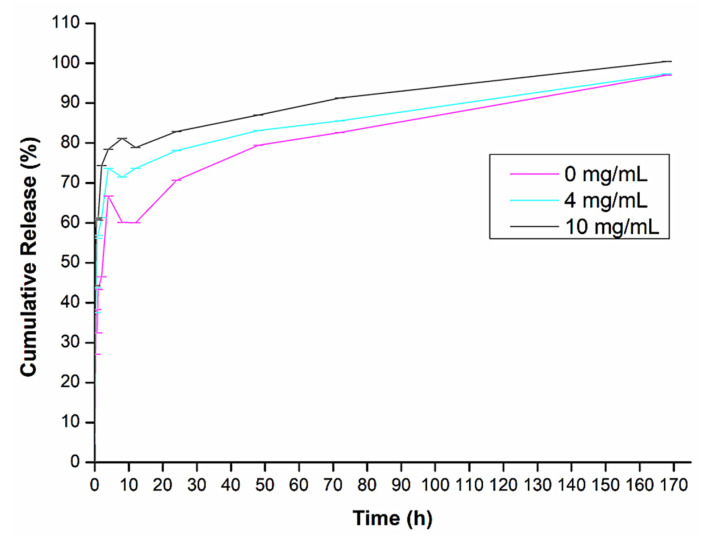
In vitro insulin release profiles from the hydrogel in solutions of differing glucose release media concentrations at 37 °C.

Insulin release mainly occurs twice: during the body’s state of fasting, i.e., normal blood glucose levels, and after eating, i.e., elevated blood glucose levels. Therefore, in this study, in vitro insulin release studies were carried out in differing glucose release media from a control (0 mg/dL glucose) to normal blood glucose levels (4 mg/dL glucose) and hyperglycemic conditions (10 mg/dL glucose) [9,15,18]. This was carried out to simulate different glycemic levels seen physiologically, to establish if the system is glucose responsive. The insulin loading efficiency within the hydrogel matrix was calculated to be 46.8%. As shown in Figure 13, the hydrogel system is indeed glucose responsive and demonstrated a sustained release over 1 week. The fluctuation over 0–10 h could be indicative of the type of insulin used (a 30/70 analogue). It is a biphasic insulin that is both rapid and long acting. Thus, the drug release curve appears to follow a biphasic pattern, the first 20 h with a faster rate of drug release and thereafter a steadier rate of drug release for the remaining time. The drug release reached a 97% after 1 week. The gel achieved sustained release of insulin in a dose-responsive manner over a period. This is in accordance with the literature demonstrating similar responsive release of insulin [42]. It should be noted that even though the fluctuation is present, the system is glucose responsive with the hyperglycemic simulated environment demonstrating the highest release of insulin. 

### 3.8. Structural Integrity of Insulin within PL–CHT–ConA Hydrogel Delivery System 

**Figure 14:**
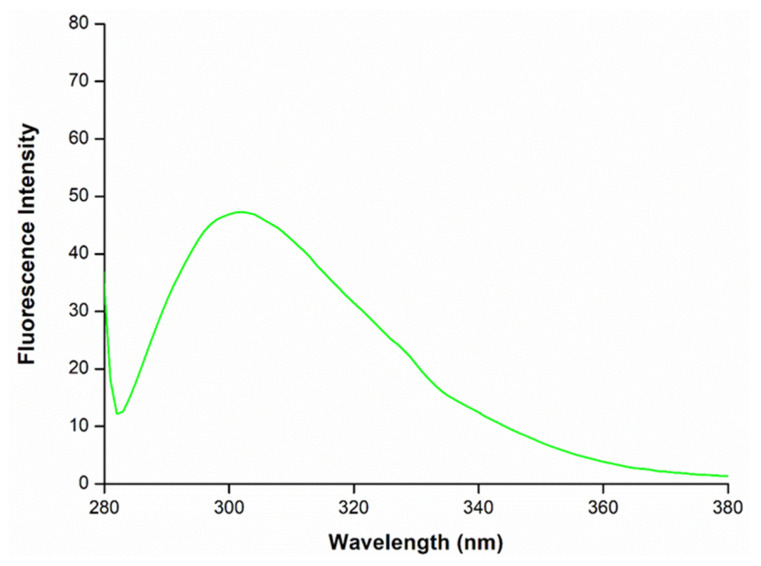
Fluorescence spectra of insulin released from the hydrogel after 7 days at 37 °C, demonstrating the structural integrity of insulin over time with the characteristic peak at around 305 nm.

As presented in the fluorescence spectrophotometry (Figure 14), the tertiary structure of insulin was observed at the characteristic peak of around 305 nm in fluorescence [8,9]. The peaks may have shifted slightly due to interference form the hydrogel system. These data suggest that the activity and the native conformation of insulin within the hydrogel over time at body temperature is conserved. 

### 3.9. Cytotoxic Assay of PL–CHT–ConA Hydrogel System

**Figure 15:**
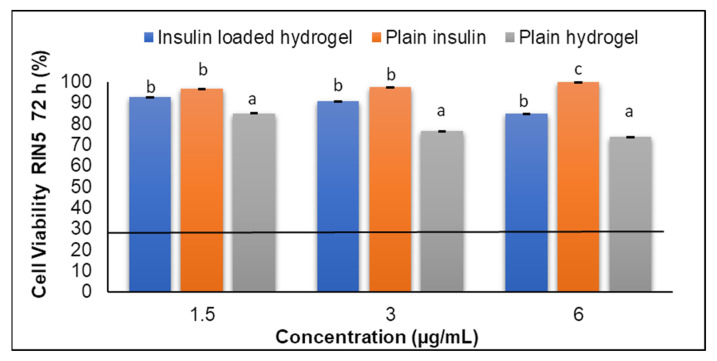
Cytotoxicity analysis carried out over 72 h on pancreatic cell lines to determine the effect of the insulin-loaded hydrogel system in comparison to commercial insulin and the unloaded hydrogel. The line indicates the viability of 5-fluorouracil as the positive control. Data are expressed as a mean ± SD (*n* = 3). a, b, c: Differing letters above the bars indicate a significant difference from other groups (Tukey’s multiple range post-hoc test, *p* < 0.05).

The viability of the insulin-loaded hydrogel system was evaluated by employing the MTT assay against RIN-5F pancreatic cell lines over 72 h (Figure 15). The insulin-loaded hydrogel, plain insulin and the unloaded plain hydrogel were studied to observe any confounding negative effects on cytotoxicity. Results demonstrated that the insulin-loaded hydrogel had a consistent viable effect on cells especially when compared to the positive control 5-fluorouracil. No negative confounding effects were observed between the loaded hydrogel, insulin and the unloaded hydrogel. Hence, the results are in accordance with previous studies [45] demonstrating no major cytotoxicity, which indicated that the formulation has good biocompatibility, as expected.

## 4. Conclusions

Several analogues of insulin exist, and, in this study, short-acting insulin was successfully delivered using the dual (glucose and thermos)-responsive hydrogel constituting covalently linked PL and CHT with ConA. PL and CHT provided enhanced viscosity and structure stability for ConA to optimally function as the glucose-responsive agent with sol–gel transitioning at pH 7.4 and 37 °C for sustained insulin delivery over 7 days. Results revealed that the stimuli-responsive hydrogel remained pharmaceutically stable and was able to perform as a closed-loop insulin delivery prototype from the insulin release data in the presence and absence of glucose as well as from the MTT assay in RIN-5F pancreatic cells. In a nutshell, the novelty of this work within the body of knowledge consists of the replacement of glucose-based polysaccharides with chitosan, which allows for preparation of chitosan–Pluronic–concanavalin A crosslinked hydrogel that enhances the effects of concanavalin A as an excellent glucose-responsive biomaterial and thereby improves the regulated release of insulin for therapeutic efficacy. This unique ConA-loaded co-forming matrix may be a promising alternative to current minimally invasive closed-loop insulin delivery systems in type 1 diabetes showing glucose responsiveness, providing sol–gel transitioning for sustaining the release of a short-acting insulin analogue over a period.

## Data Availability

The data presented in this study are all available in the manuscript.
